# Dehydration of (Perfluoroalkyl)tetramethylcyclopentenol

**DOI:** 10.3390/molecules16054031

**Published:** 2011-05-17

**Authors:** Jan Čermák, Thu Huong Nguyen Thi, Jaroslav Včelák, Alena Krupková

**Affiliations:** 1 Institute of Chemical Process Fundamentals of AS CR, v.v.i., Rozvojová 135, 165 02 Prague 6, Czech Republic; Email: vcelak@icpf.cas.cz (J.V.); krupkova@icpf.cas.cz (A.K.); 2 Department of Chemistry, Faculty of Science, Purkynje University Ústí nad Labem, České mládeže 8, 400 96 Ústí nad Labem, Czech Republic; Email: Thu-Huong.Nguyen-Thi@ujep.cz (T.H.N.T.)

**Keywords:** dehydration, fluorous cyclopentadienes, cyclopentenols

## Abstract

(Perfluoroalkyl) tetramethylcyclopentenols (alkyl = *n*-butyl, *n*-hexyl, *n*-octyl) were dehydrated to a complex mixture of endo, endo-(perfluoroalkyl) tetramethyl-cyclopentadienes and their *endo-*, *exo*-isomers. It was found in preliminary screening experiments that the best reagent for this transformation, giving an 89% yield of isomeric product mixture, was P_2_O_5_ in benzene at 80-90 °C. Products were characterized on the basis of their mass spectra and retention time information, and some peaks in the mass spectra were identified from their molecular fragments. Structures were assigned to the three most abundant products of (perfluorohexyl)tetramethylcyclopentenol dehydration. Formal dehydration kinetics showed a second order reaction in benzene but zeroth order with induction period in chlorobenzene, suggesting mass transfer limitations in the more polar chlorobenzene. Some of the products were formed by consecutive isomerization of the others, as shown by the kinetic analysis.

## 1. Introduction

The current interest in fluorous biphase systems [1] in general and in fluorous biphase catalysis [1,2,3,4,5,6,7,8] in particular has led to the introduction nowadays of a broad spectrum of ligands soluble in fluorous phases. The synthetic design of fluorous cyclopentadienes, although surprisingly somewhat lagging behind the developments, e.g. in the area of fluorous phosphines [ref. 1, p. 247], brought so far several novel types of cyclopentadienes and cyclopentadienyl complexes made fluorophilic by the attachment of polyfluorinated chains – fluorous ponytails. These include cyclopentadienes with one [9], two [10,11,12] or more [13,14] (perfluoroalkyl)ethyl chains, cyclopentadienes with silicon atoms directly bonded to the ring to separate electronic effects of the fluorous ponytail [15,16,17,18] and cyclopentadienes monosubstituted with perfluoroalkyl chains [9], which are difficult to handle or use for metal complexation. The last of the so far known types of fluorous cyclopentadienes is represented by the (perfluoroalkyl)tetramethylcyclopentadienes prepared in our group [16,19,20,21]. In the latter compounds the electrondonating effect of four methyl groups compensates for the opposite effect of the perfluoroalkyl chain bonded directly to the ring, providing stable precursors to ligands with electronic properties close to the unsubstituted cyclopentadienyl but steric properties comparable to pentamethylcyclopentadienyl. These ligands may be considered to be higher homologs of the Gassman ligand [22,23,24,25,26,27,28].

The two-step synthesis of (perfluoroalkyl) tetramethylcyclopentadienes [19] started with the nucleophilic addition of *in situ* prepared perfluoroalkyl Grignard reagent on 2,3,4,5-tetra- methylcyclopent-2-enone with the isolation of a mixture of perfluoroalkyl tertiary alcohols after aqueous acid workup. This mixture was characterized by GC-MS data only and was then subjected to dehydration with POCl_3_/pyridine [29] affording a mixture of (perfluoroalkyl)tetramethyl-cyclopentadienes and their *endo*,*exo*-isomers which were all deprotonable to only one common substituted cyclopentadienyl anion. The reported yield in this second step didn’t exceed 71%, an unsatisfactory value in view of the relatively high price of the starting compounds. Therefore, in this work the dehydration step is studied in detail, including the kinetics of formation of the diene isomers.

To our best knowledge, there are no studies dealing with dehydration of perfluoroalkyl substituted tertiary alcohols except for a few dealing with the dehydration of the first members of the homologous series, trifluoromethyl-substituted tertiary alcohols (e.g. [30,31]). Because of the expected destabilizing effect of strongly electron attracting perfluoroalkyl groups on the intermediate carbocation, our work may be of general interest to organic chemists.

## 2. Results and Discussion

### 2.1. Screening of Dehydration Agents

(Perfluoroalkyl)tetramethyl cyclopentenols were described [19] as tertiary alcohols stable at temperatures in the 70-100 °C range and resistant to dehydration by usual acid catalysis or by treatment with iodine, which are common reagents used to catalyze dehydration of tertiary alcohols. The difficulty of dehydration can be expected in view of the destabilization of intermediate tertiary carbocations by the strongly electron-accepting perfluoroalkyl group. To improve the low reported yield of (perfluoroalkyl)tetramethylcyclopentadienes we first tested several common dehydration agents including modification of the original procedure [19], Lewis acid catalysis, and use of microwave heating.

The originally used dehydration by POCl_3_ in pyridine led to partial decomposition resulting in considerable amounts of by-products (Table 1). Modification of the procedure consisting in immediate.

**Table 1:** Results of screening experiments

Reagent(s)	Solvent	Temperature [°C] /Time [h]	Sum of unreacted alcohols [%]	Sum of dienes [%]
POCl_3_	pyridine	90/4	10	59
BF_3_·Et_2_O	diethyl ether	35/9	14	77 ^b^
SOCl_2_	benzene	90/9	2	91 ^c^
PCl_5_/pyridine	benzene	90/10	0	90 ^c^
P_2_O_5_	benzene	90/9	6	89
P_2_O_5_	toluene	120/7 ^a^	5	86
P_2_O_5_ / BF_3_·Et_2_O	diethyl ether	42/8	14	83
P_2_O_5_ / BF_3_·Et_2_O	diethyl ether	25/168	10	86

^a^ microwave heating; ^b^ reaction mixture quickly decomposed; ^c^ solid by-products.

Extraction of products from the reaction mixture did not improve the procedure and only 55% yield of cyclopentadienes was obtained. Higher yield of cyclopentadienes was observed with catalysis by boron trifluoride, however the reaction mixtures gradually turned dark. Higher yields were recorded with thionyl chloride or phosphorus pentachloride in benzene, but a considerable amount of solid, probably polymeric, by-products was observed.

Reflux with phosphorus pentoxide in benzene was found to be the dehydration method of choice giving clear reaction mixtures, the lowest amount of by-products (5% as determined by GC), and only liquid by-products. Furthermore, the workup produced phosphoric acid which was likely more easily washed out from the products than HCl or BF_3_Et_2_O, thus preventing any further decomposition of product mixtures.

Our attempt to use microwave heating to accelerate the reaction in toluene solvent didn’t show any distinct improvement; boron trifluoride catalysis in addition to P_2_O_5_ treatment decreased the necessary reaction temperature even to room temperature, but also decreased somewhat the yield of cyclopentadienes. In the series of homologs the rate of reaction, followed by GC analysis of samples taken at regular intervals, changes in a surprising way: perfluorohexyl compounds dehydrate approximately two times faster than perfluorooctyl and perfluorobutyl derivatives, while perfluoro-butyl derivatives react slightly slower that perfluorooctyl compounds.

### 2.1. Kinetics of Dehydration of (Perfluoroalkyl)tetramethylcyclopentenols with P_2_O_5_

#### 2.2.1. Identification of Products

**Figure 1:**
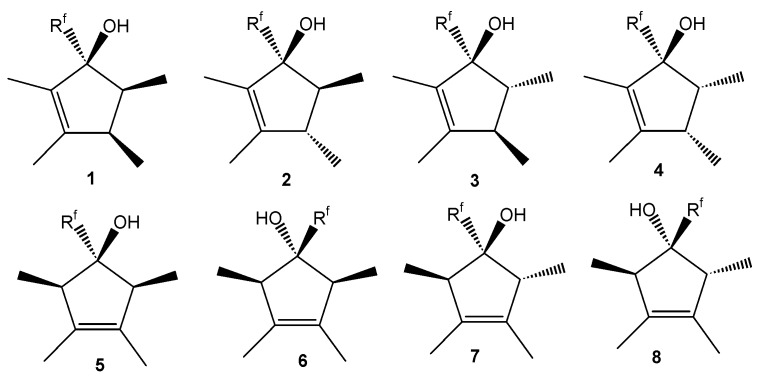
Possible structures of (perfluoroalkyl) tetramethylcyclopentenols.

**Figure 2:**
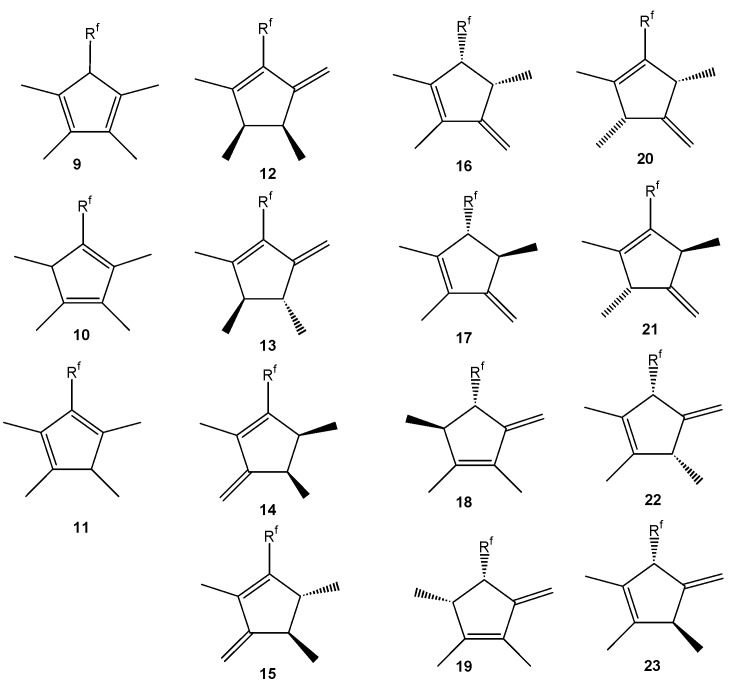
Possible structures of (perfluoroalkyl) tetramethylcyclopentadienes and their endo-, exo-isomers.

There are two posible regioisomers for starting (perfluoroalkyl) tetramethylcyclopentenols, three regioisomers of (perfluoroalkyl) tetramethylcyclopentadienes and six regioisomers of their *endo,exo*-isomers. However, these compounds may contain up to three stereocenters therefore the number of diastereomers with different physical properties is higher. The relatively high number of possible stereoisomeric structures for (perfluoroalkyl)tetramethylcyclopentenols **1**-**8**, (perfluoroalkyl)- tetramethylcyclopentadienes **9**-**11** and their *endo,exo*-isomers **12**-**23** explains the high number of individual compounds found by GC/MS (Figure 1 and Figure 2).

The identification (without generally assigning the structures) of experimentally obtained isomers **A**-**K** (dienes) and **V**-**Z** (alcohols) was based on both retention times and mass spectra, and is summarized in Table 2, Table 3, Table 4 for the three perfluoroalkyl chain lengths. The alcohols exhibited no molecular ions and the peak with the highest mass belonged to a dehydrated fragment. The fragment with *m/z* = 139 assigned to the tetramethylcyclopentenol ring after perfluorinated chain cleavage was highly characteristic for the alcohols. The dienes showed medium intensity molecular ions which then lost methyls or HF. Both alcohols and dienes contained fragments of *m/z* 171, 121, and 69, assigned to [Me_4_C_5_HCF_2_]^+^, [Me_4_C_5_H]^+^, and [CF_3_]^+^ ions, respectively. We were able to assign only structures of the three most abundant products, namely **A^6^**, which was identified as the regioisomer **12/13**, **E^6^** was identified as **9**, and the main product **G^6^** was identified as **10**, according to ^13^C-NMR spectrum analysis of samples with different proportion of individual products [19]. Interestingly, in the mass pectrum of the isomer with exocyclic double bond **A^6^** the ion with m/z = 121 prevails whereas cyclopentadiene isomers **E^6^** and **G^6^** have m/z = 171 as the basic ion. The ratio between the occurrence of ions of m/z 121 and 171 thus seems to define the type of product, either an endo,exo diene isomer or an endo,endo one.

**Table 2:** Composition of reaction mixture of perfluorobutyl derivatives ^a^.

Isomer	Type	t_Ret_ [s]	Molecular ion M^+^. (m/z)	Base peak (m/z)	Other ions (m/z)	Molar fraction at t = x min	
						0	360
**A^4^**	diene	513	340	121	325, 305, 171, 106, 91, 69	0.009	0.076
**C^4^**	diene	532	340	121	105, 93, 69	0	0.016
**D^4^**	diene	544	340	325	305, 171, 121, 91, 69	0.001	0.024
**E^4^**	diene	560	340	171	320, 305, 156, 91, 69	0.004	0.105
**F^4^**	diene	568	340	171	320, 305, 151, 91, 69	0	0.122
**G^4^**	diene	575	340	171	320, 305, 151, 91, 69	0.024	0.607
**H^4^**	diene	587		320	305, 201, 171, 151, 69	0	0.015
**I^4^**	diene	603	343	343	305, 171, 121, 91, 69	0	0.005
**V^4^**	alcohol	608		139	341,171, 121, 69	0.813	0.001
**W^4^**	alcohol	611		139	340,171, 121, 69	0.016	0
**X^4^**	alcohol	618		121	139, 95, 69	0.128	0.032

^a^ GC-MS (minor isomers omitted), glass capillary column 30 m/25 um, poly(dimethylsiloxane-co-5% methylphenylsiloxane) phase, programmed temperature: 50(5)-20/120(0)-40/250.

**Table 3:** Composition of reaction mixture of perfluorohexyl derivatives^a^.

Isomer	Type	t_Ret_ [s]	Mol. ion M^+.^ (*m/z*)	Base peak (*m/z*)	Other ions (*m/z*)	Molar fraction at t = x min			
						0^b^	483 ^b^	0 ^c^	480 ^c^
**A^6^**	diene	577	440	121	425, 171, 91, 69	0.011	0.119	0.025	0
**B^6^**	diene	581		113	403, 69, 57	0.015	0.019	0.025	0.054
**C^6^**	diene	591	440	69	121, 93	0.004	0.013	0.002	0.002
**D^6^**	diene	599	440	69	425, 171, 121, 91	0.002	0.018	0.004	0.007
**E^6^**	diene	609	440	171	420, 119, 69	0.005	0.105	0.012	0.031
**(F^6^)^d^**	diene	617	440	171	425, 151, 105, 69				
**G^6^**	diene	620	440	171	420, 201, 151, 105, 69	0.048^e^	0.679^e^	0.108^e^	0.252^e^
**H^6^**	diene	625		405	420, 186, 171, 151, 69	0.008	0.016	0	0.014
**I^6^**	diene	628		69	420, 401, 201, 181, 151, 119	0	0	0.006	0.536
**J^6^**	diene	636		420	201, 171, 151, 69	0	0	0.044	0.046
**K^6^**	diene	641	443	443	171, 121, 83, 69	0	0	0.005	0.005
**V^6^**	alcohol	646		139	440,171, 121, 69	0.818	0.031	0.582	0
**W^6^**	alcohol	648		139	440,171, 121, 69	0.09	0.026	0.11	0
**X^6^**	alcohol	655		139	443,171, 121, 95, 69	0	0.005	0.079	0.051
**Y^6^**	alcohol	658		139	440,121, 95, 69	0.005	0.005	0	0

^a^ GC-MS (minor isomers omitted), glass capillary column 30 m/25 um, poly(dimethylsiloxane-co-5% methylphenylsiloxane) phase, programmed temperature: 50(5)-20/120(0)-40/250; ^b^ solvent benzene; ^c^ solvent chlorobenzene; ^d^ not fully separated from isomer **G^6^**; ^e^ total **F^6^** + **G^6^**.

**Table 4:** Composition of reaction mixture of perfluorooctyl derivatives^a^.

Isomer	Type	t_Ret [_s]	Molecular ion M^+.^ (*m/z*)	Base peak (*m/z*)	Other ions (*m/z*)
**A^8^**	diene	641	540	121	525,171, 105, 91, 69
**B^8^**	diene	644		113	121, 69, 57
**C^8^**	diene	651	540	121	105, 91, 69
**D^8^**	diene	657	540	121	525, 171, 105, 69
**F^8^**	diene	666	540	171	521, 156, 121, 105, 69
**G^8^**	diene	673	540	171	520, 201, 151, 69
**H^8^**	diene	679	540	201	520, 501, 171, 151, 69
**V^8^**	alcohol	694		139	540,121, 69
**W^8^**	alcohol	696		139	540,121, 69
**X^8^**	alcohol	702		139	540,121, 105, 95, 69
**Y^8^**	alcohol	706		139	540,121, 95, 69
**Z^8^**	alcohol	710		139	540,157, 113, 95, 69,56

^a^ GC-MS (minor isomers omitted), glass capillary column 30 m/25 um, poly(dimethylsiloxane-co-5% methylphenylsiloxane) phase, programmed temperature: 90(5)-20/120(0)-40/250.

#### 2.2.2. Kinetic Observations

Reaction with P_2_O_5_ at 80 °C in benzene and the more polar chlorobenzene (perfluorohexyl derivatives only) was chosen for the formal kinetic evaluation of the dehydration of (perfluoroalkyl)- tetramethylcyclopentenols. The chromatographic separation conditions were adjusted to maximum possible separation of individual isomers (see Table 2, Table 3, Table 4). The dehydration of the sum of alcohols to the sum of dienes formed the input of the kinetics treatment. 

**Figure 3:**
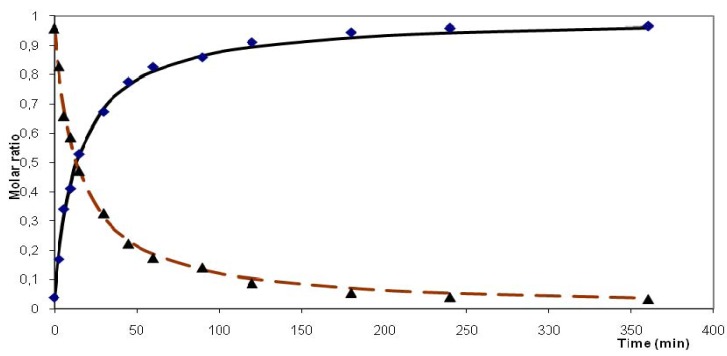
Dehydration of (perfluorobutyl) tetramethylcyclopentenols (P_2_O_5_, benzene, 80 °C) k = 0.071, n = 2, s.d. = 0.017; points-experimental concentrations, lines-fitted functions; solid line-dienes total, dashed line-alcohols total.

Figure 3 shows the concentration changes of both alcohols and dienes with reaction time. The experimental data were fitted obtaining thus the estimates of rate constants *k* and reaction orders *n* together with the estimated standard deviation *s.d.* of the fitting. Our own program for fitting formal kinetics was based on the parametric nonlinear optimization method designed by Marquardt [32].

**Figure 4:**
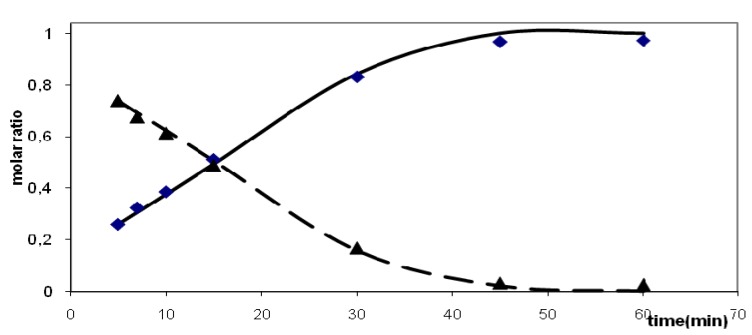
Dehydration of (perfluorohexyl) tetramethylcyclopentenols (P_2_O_5_, chlorobenzene, 80 °C) individual reaction of alcohol isomer **V^6^** into the dienes isomers **F^6^** **+** **G^6^** **+** **E^6^** k = 0.023, n = 0, s.d. = 0.023, induction period 5min; points-experimental concentrations, lines-fitted functions; solid line-dienes **F^6^** **+** **G^6^** **+** **E^6^**, dashed line-alcohol **V^6^**.

The decrease of an individual alcohol (or group of alcohols) concentration and increase of an individual diene (or a small group of dienes) concentration with time was taken as a basis for assignment of particular dienes being products of particular alcohols dehydration. Dehydration of both perfluorobutyl and perfluorohexyl substituted alcohols in benzene apparently follows a second order with respect to the starting alcohol. Dehydration of the perfluorohexyl substituted alcohol in chlorobenzene followed a different course and the reaction was better described as formally being of zeroth order with an induction period of 5 min (Figure 4). 

Such kinetics can often be found in reaction systems with mass transfer of one of the reactants through phase boundary as a rate limiting step (Table 5). Formation of microemulsions was suggested in apparent solutions of fluorous compounds in organic solvents.

**Table 5:** Kinetic approximation of dehydration of (perfluoroalkyl)cyclopentenols.

Substrate	Solvent	Temp. (°C)	Rate constant^a^	Reaction order	Standard deviation	Time_50 %_^c^ [min]	Time_90 %_^c^ [min]
**CPT-C6F13-OH**	benzene	80					
total reaction			0.022	2	0.042	36	385
individual reaction: **V^6^** to **F^6^+G^6^+E^6^**			0.036	2	0.032		
**CPT-C6F13-OH**	Cl-benzene	80					
total reaction			0.016	0	0.085	15	38
individual reaction: **V^6^** to **F^6^+G^6^+E^6^**			0.023	0^b^	0.023		
**CPT-C4F9-OH**	benzene	80					
total reaction			0.071	2	0.017	14	114
individual reaction: **V^4^+X^4^** to **F^4^+G^4^**			0.056	2	0.086		

^a^ concentration in molar fractions, time in [min]; ^b^ induction period 5 min; ^c^ time to reach 50% and 90% total yield of dienes, respectively.

The analysis of time dependence of individual perfluorobutyl substituted diene isomers revealed that the major alcohol isomer (81% in the starting mixture, denoted **V^4^**) was transformed into a diene mixture comprising major diene isomers **F^4^** and **G^4^**. The second most abundant alcohol **X^4^** (13% in the starting mixture) seemed to be the main source for diene **A^4^**; both compounds were characterized by the presence of an ion with *m/z* = 121 in the mass spectra. The rate of formation of diene isomer **E^4^** exceeded the overall rate of alcohol dehydration, especially above 90% conversion, which is evidence for diene **E^4^** being formed by the isomerization of other diene isomers.

**Figure 5:**
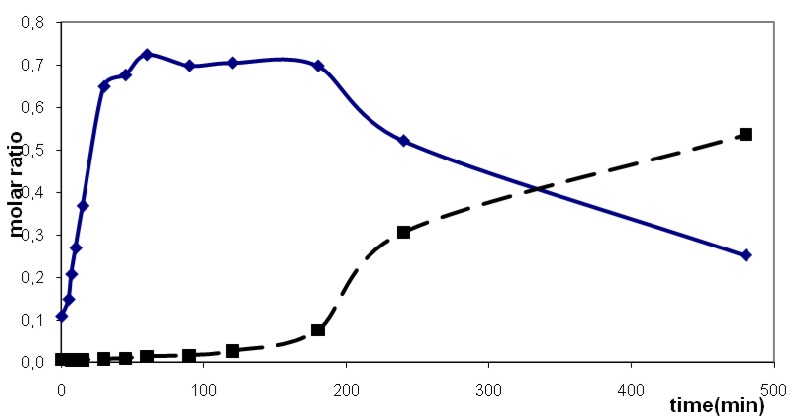
Dehydration of (perfluorohexyl)tetramethylcyclopentenols (P_2_O_5_, chloro-benzene, 80 °C). Isomeration of (perfluorohexyl)tetramethylcyclopentadienes **F^6^** **+** **G^6^** into isomer **I^6^** solid line-generation of diene isomers **F^6^** **+** **G^6^**, dashed line-generation of diene isomer **I^6^**.

Dehydration of perfluorohexyl substituted alcohol isomers involved the reaction of isomer **V^6^** mainly (82% in the starting mixture) giving dienes **F^6^** and **G^6^** together with isomer **E^6^**. The different rate of formation of isomer **A^6^** supports its formation by isomerization from the other dienes. 

The course of dehydration of C_6_ alcohol in chlorobenzene differed somewhat from that found in benzene. In chlorobenzene, the yield of the major product, i.e. the dienes **F^6^** **+** **G^6^** reached a maximum at 180 min reaction time, at longer times the dienes seemed to isomerize to diene **I^6^** (Figure 5). Similarly, the concentration of isomer **A^6^** went through a maximum in the course of the reaction, the amount of **A^6^** being outside detection limit already at 240 min reaction time.

## 3. Experimental

### 3.1. General

Manipulations with Grignard reagents were carried out under an argon atmosphere. Perfluorohexyl and perfluorooctyl iodide (both ABCR), perfluorobutyl iodide, phosphorus oxychloride and chlorobenzene, (all Aldrich), bromobenzene (Latwopalne) and the solvents benzene, toluene, and pyridine (all Lachema) were commercial products used without further purification. (Perfluoroalkyl)- tetramethylcyclopentenols (mixtures of isomers) were prepared according to the literature method [19]. Preliminary dehydration experiments were analyzed by gas chromatography on a Hewlett Packard 5890 instrument using nitrogen carrier gas with HP DB-5 column (30 m × 0.25 mm, 5% phenylmethylsilicone stationary phase). Temperature program: 100 °C (5 min)-5 °C/min-280 °C final. ^13^C{^1^H} NMR spectra were measured on a Varian Mercury 300 spectrometer at 75.44 MHz in or CDCl_3_ solutions at 25 °C. Chemical shifts (δ) are reported in ppm relative to TMS, referenced to the solvent peak.

### 3.2. Screening Dehydration Experiments-General Procedure

The experiments were carried out with 3 mmol of the mixture of (perfluoroalkyl) tetramethyl-cyclopentenols in 10 mL of solvent unless stated otherwise, and progress of reactions was followed by gas chromatography at 1-2 h intervals. The reaction mixture was treated with ice or ice-cooled water, organic layer separated, aqueous layer extracted with ether (3 × 10 mL), the combined organic leyers were washed with saturated NaHCO_3_ solution (20 mL) and with saturated NaCl solution (20 mL) then dried overnight with MgSO_4_. The mixtures were then distilled, solvents were removed under atmospheric pressure and products collected in vacuum: fraction boiling at 65-67 °C/5 Torr for perfluorobutyl, 83-85 °C/4 Torr for perfluorohexyl, and 102-103 °C/3 Torr for perfluoorooctyl derivatives.

### 3.3. Dehydration with POCl_3_

Phosphorus oxychloride (1.6 mL, 17 mmol) was cooled to −5 °C, pyridine (6 mL) added and the solution of (perfluorobutyl)tetramethylcyclopentenols (1.074 g, 3 mmol) in pyridine (11 mL) slowly dropped to the mixture under stirring and cooling to 0 °C. The reaction mixture was then heated at 90 °C for 4 h, then worked up as mentioned above. The product (0.95 g) contained 59% of dienes (GC yield 55%), 10% of unreacted alcohols and 28% of unidentified by-products.

### 3.4. Dehydration with BF_3_·Et_2_O

Boiling of (perfluorobutyl)tetramethylcyclopentenols (1.074g, 3 mmol) with equimolar amount of BF_3_·OEt_2_ (0.4 mL) in diethyl ether (10 mL) gave after 9 h 1.091 g of the product containing 77% of dienes (GC yield 82%), 14% of unreacted alcohols and 9% of unidentified by-products.

### 3.5. Dehydration with SOCl_2_

Boiling of (perfluorobutyl)tetramethylcyclopentenols (1.074 g, 3 mmol) with equimolar amount of thionyl chloride (0.22 mL) in benzene (10 mL) gave after 10 h 0.923 g of the product containing 91% of dienes (GC yield 82%), 2% of unreacted alcohols and 7% of unidentified by-products.

### 3.6. Dehydration with PCl_5_

Boiling of (perfluorobutyl)tetramethylcyclopentenols (1.074g, 3 mmol) with half of the equimolar amount of phosphorus pentachloride (0.32 g in 0.97 mL of pyridine) in benzene (10 mL) gave after 10 h 0.930 g of the product containing 44% of dienes, 53% of unreacted alcohols and 3% of unidentified by-products.

Using equimolar amount of phosphorus pentachloride afforded 0.915 g of the product containing 90% of dienes (GC yield 81%), no unreacted alcohols and 10% of unidentified by-products.

### 3.7. Dehydration with P_2_O_5_ in Benzene

(Perfluorobutyl) tetramethylcyclopentenols (1.074 g, 3 mmol) were added to the suspension of P_2_O_5_ (0.4 g, 2.8 mmol) in benzene (10 mL) with stirring. The reaction mixture was then heated at 90 °C for 9 h, then worked up as mentioned above. The product (0.92 g) contained 89% of dienes (GC yield 80%), 6% of unreacted alcohols and 5% of unidentified by-products.

### 3.8. Dehydration with P_2_O_5_ in Toluene (Microwave Heating)

(Perfluorobutyl) tetramethylcyclopentenols (1.074 g, 3 mmol) were added to the suspension of P_2_O_5_ (0.25 g, 1.75 mmol) in toluene (10 mL) with stirring. The reaction mixture was then refluxed in a microwave reactor at 25% of the full power (i.e. 150 W) for 7 h. The product (0.96 g) contained 86% of dienes (GC yield 81%), 5% of unreacted alcohols and 9% of unidentified by-products.

### 3.9. Dehydration with P_2_O_5_ in the Presence of BF_3_·Et_2_O at 40 °C

The mixture of phosphorus pentoxide (0.852 g, 6 mmol) and (perfluorobutyl) tetramethyl-cyclopentenols (1.074 g, 3 mmol) in diethyl ether (10 mL) was cooled to −5 °C and BF_3_·Et_2_O (40 μL, 0.045 g, 0.317 mmol) added with stirring. The reaction mixture was then heated at 42 °C for 8 h, then worked up as mentioned above. The product (0.921 g) contained 83% of dienes (GC yield 75%), 14% of unreacted alcohols and 3% of unidentified by-products.

### 3.10. Dehydration with P_2_O_5_ in the Presence of BF_3_·Et_2_O at Room Temperature

The same mixture as prepared above was stirred at room temperature for 7 days. The product (0.819 g) contained 86% of dienes (GC yield 69%), 10% of unreacted alcohols and 4% of unidentified by-products.

### 3.11. Kinetics

Samples of reaction mixtures were filtered through K_2_CO_3_ to remove P_2_O_5_ and diluted by diethyl ether. GC-MS analysis was run on a Varian 3500 gas chromatograph equipped with a glass capillary column (30m length and 0.025 mm diameter) coated with poly(dimethylsiloxane-co-5% methylphenylsiloxane) phase (temperature program 50-250 °C) connected to a Finnigan Mat mass ITD detector working at 70 eV. The acquisition range was 45 to 400, 500 or 600 a.m.u., according to the length of perfluoroalkyl chain. In the course of dehydration reaction to the mixture of isomeric (perfluoroalkyl)tetramethylcyclopentadienes, changes of the isomer composition of starting alcohols mixture were also monitored. Unfortunately, some of the isomers of starting alcohols as well as isomers of dienes were not always separable.

### 3.12. NMR Characterization of Identified Main Products

#### Isomer **9**

^13^C-NMR (CDCl_3_, 125.7 MHz): 10.94 s (CH_3_); 12.48 s (CH_3_); 60.27 t, ^2^J_C-F_ = 17.5 (*C*-CF_2_); 128.01 s (>C=); 140.07 s (>C=); 108-121 m (CF).

#### Isomer **10**

^13^C-NMR (CDCl_3_, 125.7 MHz): 10.36 s (CH_3_); 11.67 s (CH_3_); 12.74 bs (CH_3_); 14.00 bs (CH_3_); 50.06 s (CH); 127.49 t, ^2^J_C-F_ = 23.4 (=*C*-CF_2_); 134.28 d, ^3^J_C-F_ = 1.8 (>C=); 146.49 s (>C=); 150.83 bs (>C=); 108-121 m (CF).

#### Isomers **12/13**

^13^C-NMR (CDCl_3_, 125.7 MHz): 11.11 s (CH_3_); 20.39 s (CH_3_); 20.49 s (CH_3_); 45.73 s (CH); 47.38 s (CH); 106.31 s (=CH_2_); 145.54 bs (>C=); 159.04 s (>C=); 108-121 m (CF).

## 4. Conclusions

Dehydration of (perfluoroalkyl) tetramethylcyclopentenols gave a broad spectrum of products which were isomers of either *endo,endo*- or *endo,exo*-cyclopentadienes. The reagent of choice for the dehydration was phosphorus pentoxide in benzene with which the yield was improved up to 89% of dienes in comparison with the literature. Structures could be assigned to three main products of the dehydration showing that the *endo,endo*-isomers prevail. Due to complexity of reaction mixture, formal kinetic evaluation of the dehydration reaction was carried out with sum of alcohols on one side of the reaction and sum of dienes on the other side, some products were followed independently, however. In benzene, perfluorobutyl and perfluorohexyl substituted alcohols followed the second order dehydration kinetics while in more polar chlorobenzene perfluorohexyl substituted alcohol followed the zeroth order reaction with an induction period suggesting mass transfer limitations. At higher conversions some of the products isomerized, the reaction being more pronounced in chlorobenzene.
